# Two is not always better than one: ST-segment elevation myocardial infarction and acute pulmonary embolism in COVID-19

**DOI:** 10.1093/omcr/omac056

**Published:** 2022-06-23

**Authors:** Avinash Radhakrishna, Mohammed Omar Abdelaziz, Niall Mulvihill

**Affiliations:** Department of Cardiology, St. Vincent’s University Hospital, Dublin 4, Ireland; Department of Cardiology, St. Vincent’s University Hospital, Dublin 4, Ireland; Department of Cardiology, St. Vincent’s University Hospital, Dublin 4, Ireland

## Abstract

Coronavirus disease 2019 (COVID-19) has become a significant global health burden with potential consequences on the cardiovascular system. We describe a case of a healthy male with no prior cardiovascular risk factors who developed ST-segment elevation myocardial infarction and pulmonary embolism following a diagnosis of severe COVID-19 pneumonitis. The patient made a significant recovery following coronary thromboaspiration and anticoagulation therapy. Multiple mechanisms including an indirect hyperinflammatory immune response and/or direct endothelial damage may explain the prothrombotic state related to COVID-19. The cytokine storm leads to endothelial dysfunction and subsequent thromboembolism. Awareness of the lethal cardio-pulmonary sequalae of COVID-19 is important as surges continue across the world owing to new variants.

## INTRODUCTION

Coronavirus disease-2019 (COVID-19) caused by the novel coronavirus, severe acute respiratory syndrome coronavirus-2 (SARS-CoV-2) was first described in December 2019, in Wuhan [1]. The outbreak which started locally in China has spread rapidly across the globe, drawing the attention of cardiologists worldwide because of its association with cardiovascular diseases. The exact mechanism of cardiovascular involvement is not clearly understood but those with underlying cardiovascular disease and/or risk factors including male sex, advanced age, diabetes mellitus, hypertension and obesity are predisposed to COVID-19 and are at a greater risk of developing adverse cardiovascular outcomes [1]. We herein present a case of a 54-year-old man with no underlying cardiovascular risks who developed ST-elevation myocardial infarction and pulmonary embolism (PE) following a diagnosis of severe COVID-19 pneumonitis.

## CASE REPORT

A 54-year-old gentleman of African origin with no underlying medical health issues presented to the hospital due to worsening shortness of breath and productive cough over 5 days. His respiratory symptoms were associated with persistent chills, rigors and generalized myalgia. On arrival, his arterial blood gas showed type 1 respiratory failure requiring 40% fraction of inspired oxygen (FiO_2_) to maintain saturations. He was tachypnoeic (respiratory rate of 32 breaths/min), tachycardic (heart rate of 115 beats/min) and pyrexic at 38.2°C. His blood pressure was 112/54 mmHg after 1 L bolus of intravenous fluids.

**Figure 1:**
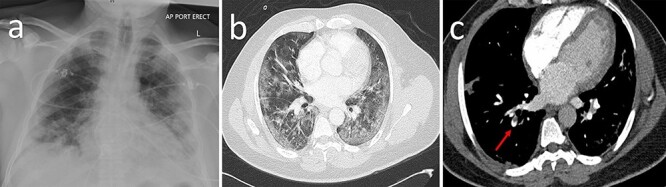
Pulmonary disease in COVID-19. (**a**) Chest X-ray demonstrating severe bilateral patchy infiltrates. (**b**) Extensive subpleural and peribronchovascular ground-glass infiltrates on computed tomography. (**c**) Filling defect in the right lower pulmonary subsegmental artery on CT pulmonary angiography (red arrow).

Clinical examination revealed the patient visibly breathless with increased respiratory effort and bilateral coarse inspiratory crackles throughout both lung fields. His chest radiograph showed extensive bilateral patchy airspace opacification (Fig. 1**a**), but the admission electrocardiography (ECG) was unremarkable. Blood tests revealed a significantly elevated C-reactive protein (CRP) of 415 mg/L (normal range 0–5) and Troponin-T was normal.

As there was a high clinical suspicion for COVID-19, a reverse transcription-polymerase chain reaction was performed which confirmed the diagnosis. He was then commenced on Dexamethasone 6 mg, prophylactic enoxaparin (4000 IU daily) and transferred to the designated COVID ward for further care. On the fourth day of admission, he unexpectedly developed pleuritic chest pains and worsening breathlessness with an increase in FiO_2_ of 50%. D-Dimer was mildly elevated at 0.73 μg Fibrinogen Equivalent Units (FEU)/ml (0–0.5) which warranted a Computed Tomography Pulmonary Angiography (CTPA). The CT scan showed no evidence of PE but there was extensive subpleural and peribronchovascular ground-glass infiltrates throughout the lungs which were typical features of COVID-19 pneumonitis (Fig. 1**b**).

**Figure 2:**
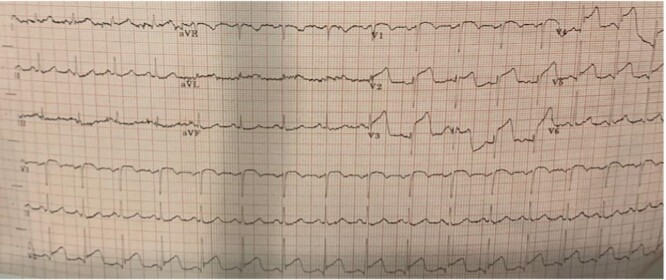
Electrogram showing ST-segment elevations in leads v1–v5 with evolving ST-segment changes in leads I and aVL.

The patient appeared to be on course for recovery until Day 8 of admission when he suddenly developed central crushing chest pains which woke him from sleep at 7 a.m. ECG revealed marked ST-segment elevations in leads v1–v5, consistent with an acute anterior ST-segment elevation myocardial infarction (STEMI) (Fig. 2). Troponin-T was markedly raised at 750 ng/L (range 0–14) and 6 h later increased to 8432 ng/L. He was loaded with dual antiplatelet therapy (DAPT) and was urgently transferred to the catheterization laboratory.

**Figure 3:**
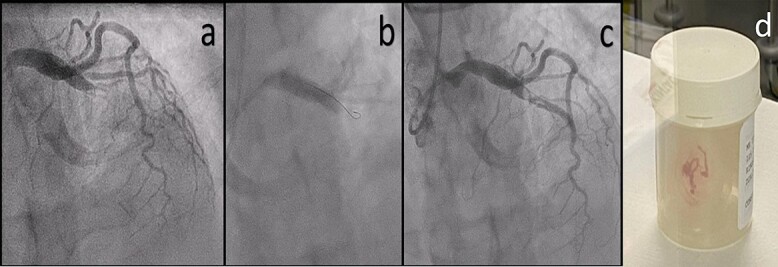
(**a**) Coronary angiogram showing an acute occlusion of the mid left LAD. (**b**) Crossing the lesion smoothly with a Sion Blue wire. (**c**) Multiple large thrombi in the mid LAD section. (**d**) Fresh red thrombus after thromboaspiration.

Coronary angiogram revealed a single-vessel, total mid left anterior descending artery (LAD) occlusion (Fig. 3**a**). All other vessels were normal. With support using an EBU 4 guide catheter, the acute lesion was successfully crossed using a 0.014×190 cm Sion Blue wire (Fig. 3**b**). The mid-LAD was carefully dilated with a 3.5×20 mm NC Trek balloon and eventually after multiple attempts, there was minimal flow into the LAD with multiple filling defects, suggestive of acute thrombus formation (Fig. 3**c**). An aspiration catheter was used to extract large amounts of red thrombus (Fig. 3**d**). Despite repeated balloon dilatations, flow in the distal LAD remained poor. The *glycoprotein IIb*/*IIIa* inhibitor, eptifibatide and a bolus dose of unfractionated heparin (5000 IU) were given.

Transthoracic echocardiography revealed severe hypokinesia of the apex and anterior wall with moderately impaired left ventricular systolic function. Spontaneous echo contrast was also noted in the left ventricular cavity. At this point, the patient was mobilizing on the wards while on prophylactic enoxaparin (4000 IU daily) and DAPT. Four days later, he started experiencing pleuritic chest pains in the context of a markedly raised D-Dimer of 4.24 μg FEU/ml (0–0.5). This time, CTPA confirmed a PE (Fig. 1**c**) and Rivaroxaban 20 mg was commenced alongside Clopidogrel 75 mg and Aspirin 75 mg daily. Screening for cardiovascular risk factors and hypercoagulability was negative, but ferritin, interleukin-6, lactate dehydrogenase and CRP levels were markedly raised, suggesting a hyperinflammatory state. The patient’s overall clinical condition gradually improved and was discharged after 18 days of hospitalization with follow-up in the COVID-19 clinic, cardiology clinic and cardiac rehabilitation services.

## DISCUSSION

Previous reports of cardio-pulmonary thromboembolism in COVID-19 have patients with underlying cardiovascular risk factors, primarily diabetes mellitus which is commonly associated with preexisting coronary artery disease and diabetic cardiomyopathy that is frequently undiagnosed owing to its asymptomatic nature [2–4]. We describe an uncommon case of cardio-pulmonary thromboembolism in an acute COVID-19 patient without any underlying medical issues.

In the literature, there are several theoretical mechanisms linking COVID-19 with atherothrombosis. During an acute infection, enzymes released from activated macrophages break down collagen, which is a major component of the fibrous cap found on atherosclerotic plaques, leading to plaque instability. Proinflammatory macrophages then release tissue factor which triggers the coagulation cascade causing hypercoagulability and thrombus formation [5].

Patients with severe COVID-19 pneumonitis are in a hyperinflammatory and prothrombotic state due to the increased production of inflammatory cytokines and thrombotic markers such as fibrinogen and D-Dimer. The cytokine storm produced triggers platelet activation, thrombin formation and finally multiorgan thrombosis [6]. Angiotensin-converting enzyme 2 (ACE2) receptors facilitates SARS-CoV-2 entry into human cells and since ACE2 receptors are widely expressed in arterial endothelial cells, direct vascular injury caused by COVID-19 may lead to endothelial dysfunction and intraluminal thrombosis. These findings are in keeping with histological studies by Varga et al., which showed the presence of SARS-CoV-2 material within endothelial cells and accumulation of inflammatory cells with evidence of cell necrosis [7].

A timely reperfusion strategy should be the mainstay of treatment for STEMI and management protocols should be tailored to suit regional heterogeneity in healthcare resources. The latest European Society of Cardiology guidelines recommend primary Percutaneous Coronary Intervention (PCI) as the reperfusion therapy of choice if performed within 120 min and may be delayed up to an additional 60 min due to logistic reasons [8]. The Society for Cardiovascular Angiography and Interventions and other expert groups have recommended the prompt use of fibrinolytic therapy in selected patients with STEMI if delays are inevitable [8, 9]. Observational studies have demonstrated that COVID-19 patients have a greater thrombus burden with higher rates of stent thrombosis, requiring aspiration thrombectomy and *glycoprotein IIb*/*IIIa* inhibitor [10].

As we enter a new phase of the pandemic, further research must continue to improve our understanding of the viral–host interaction to help guide effective therapies, especially the role of anticoagulation in COVID-19. Although there is a broad differential diagnosis for myocardial injury, awareness of the lethal cardio-pulmonary sequalae of COVID-19 is important as surges continue across the world owing to new variants.
